# A glance on the role of actin in osteogenic and adipogenic differentiation of mesenchymal stem cells

**DOI:** 10.1186/s13287-020-01789-2

**Published:** 2020-07-16

**Authors:** Asmat Ullah Khan, Rongmei Qu, Tingyu Fan, Jun Ouyang, Jingxing Dai

**Affiliations:** Guangdong Provincial Key Laboratory of Medical Biomechanics, Department of Anatomy, School of Basic Medical Science, Southern Medical University, Guangzhou, China

**Keywords:** Mesenchymal stem cells (MSCs), Actin, Osteogenesis, Adipogenesis cytoskeleton, Osteogenic differentiation, Adipogenic differentiation, Cytoskeleton

## Abstract

Mesenchymal stem cells (MSCs) have the capacity to differentiate into multiple lineages including osteogenic and adipogenic lineages. An increasing number of studies have indicated that lineage commitment by MSCs is influenced by actin remodeling. Moreover, actin has roles in determining cell shape, nuclear shape, cell spreading, and cell stiffness, which eventually affect cell differentiation. Osteogenic differentiation is promoted in MSCs that exhibit a large spreading area, increased matrix stiffness, higher levels of actin polymerization, and higher density of stress fibers, whereas adipogenic differentiation is prevalent in MSCs with disrupted actin networks. In addition, the mechanical properties of F-actin empower cells to sense and transduce mechanical stimuli, which are also reported to influence differentiation. Various biomaterials, mechanical, and chemical interventions along with pathogen-induced actin alteration in the form of polymerization and depolymerization in MSC differentiation were studied recently. This review will cover the role of actin and its modifications through the use of different methods in inducing osteogenic and adipogenic differentiation.

## Introduction

Stem cells exhibit a great potential for use in tissue engineering because of their regenerative capacity in many tissues, including nervous tissue, muscle tissue, adipose tissue, cartilage tissue, and bone tissue. Examples of cells with this potential include embryonic stem cells, induced pluripotent cells, mesenchymal stem cells (MSCs), and hematopoietic stem cells. Mesenchymal stem cells are multipotent, meaning that they can differentiate into numerous cell types, and in particular, adipocytes, chondrocytes, and osteocytes [1]. The cytoskeleton is known to play a crucial role in the differentiation of MSCs; however, this review will focus specifically on the role of actin.

Actin is a globular protein with a molecular weight of approximately 42 kDa and consists of four structural domains [2]. Actin exists in two forms, monomeric G-actin and filamentous actin (F-actin). The filamentous form is considered to be crucial for the structure of the cytoskeleton. Actin filament organization leads to the formation of fiber bundles or three-dimensionally structured networks. These fiber bundles help maintain mechanosensing and mechanotransduction [3–5] which eventually allow cells to migrate, proliferate, and differentiate. In addition, the execution of cell movement is achieved through the formation of lamellipodia with the help of densely branched actin filaments. In addition, actin forms sensory structures in the form of filopodia, which facilitates signal transduction. Actin polymerization and stress fiber formation are essential for the interaction between cells and the extracellular matrix (ECM) [6].

Various cues including chemical, mechanical stress, nanomaterials, and pathogen affect the process of actin polymerization which will be discussed in detail in the review.

## Actin properties

### The effect of actin on cell shape and cell spreading

The actin cytoskeleton is a crucial determinant of cell shape, which can be more precisely explained as the assembly and disassembly of actin filaments [7–9]. Notably, various biological processes such as proliferation [10] and differentiation [11, 12] are influenced by cell shape. In addition, actin cytoskeleton-mediated cell shape changes have been shown to be vital for the regulation of MSC lineage commitment [13].

**Fig. 1:**
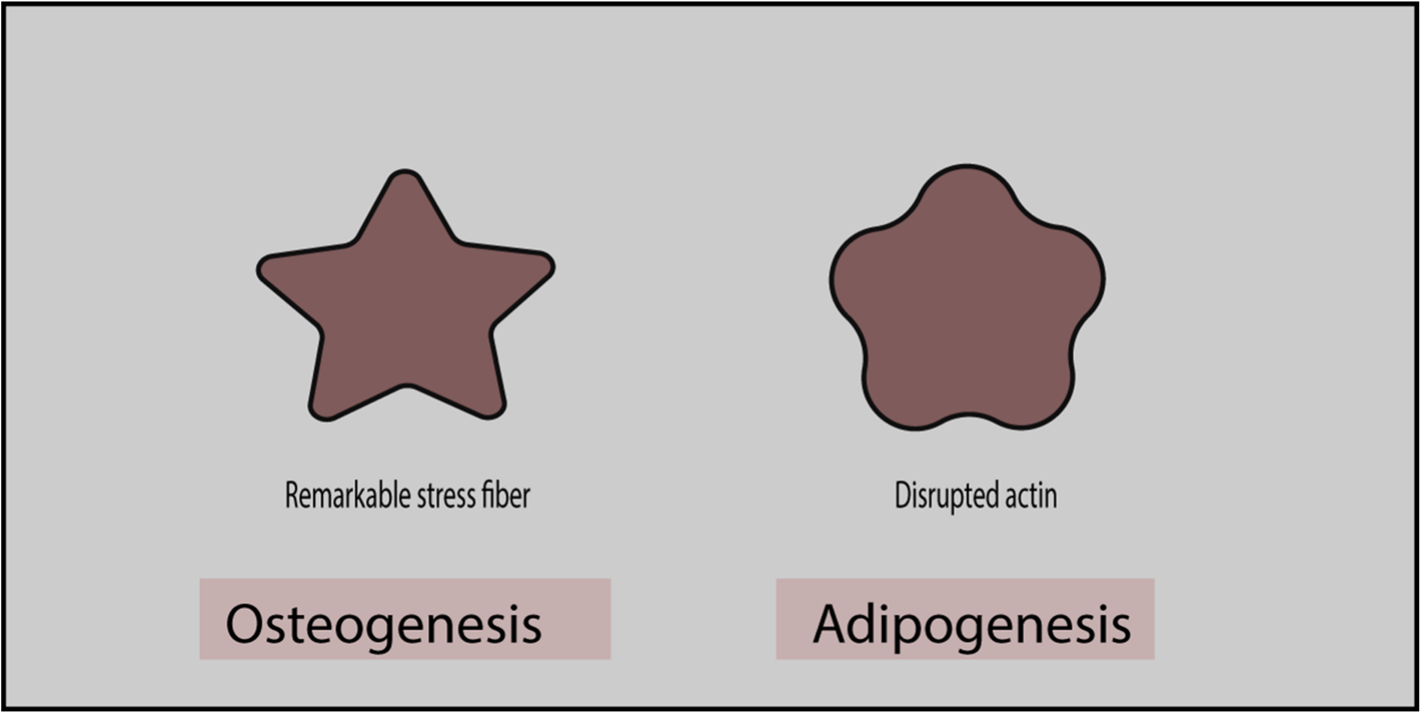
Star-shaped and flower-shaped cells that favor osteogenesis and adipogenesis, respectively

Several studies have reported the influence of the actin cytoskeleton and cell shape on MSC differentiation, wherein MSCs exhibit a flower shape during adipogenic differentiation and a star shape during osteogenic lineage commitment [14, 15]. In addition, a high-stress fiber density can clearly be observed in star-shaped cells, whereas flower-shaped cells present disrupted actin filaments (Fig. 1) [15]. Similarly, scanning electron microscopy revealed that adipogenic cells adopt round-shaped forms, whereas angular form containing more projections is common in osteogenic cells. In contrast, undifferentiated cells elongate into spindle-shaped cells. Increased actin polymerization with perinuclear actin bundles framing the nucleus is observed during osteogenesis, whereas a disrupted actin network is observed during adipogenesis [16].

Cell shape directly mediates Rho (Ras homologous) activity which leads to the activation of Rho-associated coiled-coil-containing protein kinase (ROCK), hence creating cell tension by phosphorylating myosin light chain (MLC). Force generated by actomyosin contraction determines the shape of cells which eventually affect the stem cell fate [17]. Similarly, Rho activity was reported to be higher in cells that spread properly than in unspread cells. Moreover, when cells are grown at a high-density place, they differentiate along the adipogenic cell lines, and in contrast to that, osteogenic cell lines are adopted at low-density sites. This difference can be explained by the fact that they have different cell spreading and cell shape [17]. In addition, when a micropattern substrate was used to investigate the importance of cell spreading in osteogenesis, the results showed that osteogenesis was favored when cells could spread over a large area, whereas adipogenesis was facilitated when cell spreading occurred over a smaller area [17, 18].

The actin cytoskeleton was recently reported to influence the shape of the nucleus during adipogenic and osteogenic differentiation [19]. Adipogenic differentiation leads to a decrease in the size of the nucleus, likely because of a disruption in the actin filament structure [20]. In contrast, F-actin polymerization increases the size of the nucleus during osteogenic differentiation [21]. Actin perinuclear cap, made of contractile bundles, is connected to focal adhesion with nucleus through the LINC complex. This physical connection between focal adhesion and nucleus is crucial for force transmission to the nucleus [22–24]. Stiffness of the nucleus is also affected by actin polymerization as actin polymerization increases nuclear stiffness, whereas actin depolymerization decreases nuclear stiffness [25]. However, further studies are required to understand actin’s role in nuclear mechanics and how nuclear mechanics and nuclear morphology affect stem cell differentiation.

### Mechanical properties of actin

The actin cytoskeleton regulates the mechanical behavior of cells through its assembly and disassembly. Specifically, cell stiffness is influenced by the F-actin cytoskeleton. Extensive modulation of actin filaments occurs when cells undergo differentiation [26]. Disrupting F-actin results in the cells to be softer and more viscous than control cells. Actin-disrupting drugs induce the degradation of actin filaments, which reduces actin density at the cortical region and eventually renders cells softer [27, 28]. Thin and long actin filaments extend parallel to the long axis in undifferentiated or control cells, whereas the actin cytoskeleton reorganizes into a disrupted meshwork around the oil droplet in cells undergoing adipogenic differentiation [29, 30]. Osteogenic cells present comparatively thick bundled fibers at the periphery. Early studies regularly reported that osteoblasts are comparatively stiffer than adipocytes [31, 32].

Various techniques have been developed to evaluate the mechanical behavior of cells, including microaspiration, atomic force microscopy (AFM), nanoindentation, optical tweezers, and force traction microscopy [33]. A micropipette was used to measure the elastic modulus before and after MSC differentiation, and differentiated stem cells were reported to be stiffer than undifferentiated MSCs [34]. Young’s modulus during osteogenic differentiation is 0.6-fold higher than that during adipogenesis; however, Young’s moduli of MSCs decreased significantly after treatment with cytochalasin D (CD) [31]. Actin filaments are thicker in MSCs than in osteoblasts, which present a thin and dense actin network. Thick actin filaments render Young’s modulus of MSCs higher than that of osteoblasts [35].

## Actin cytoskeleton and Rho pathway

**Fig. 2:**
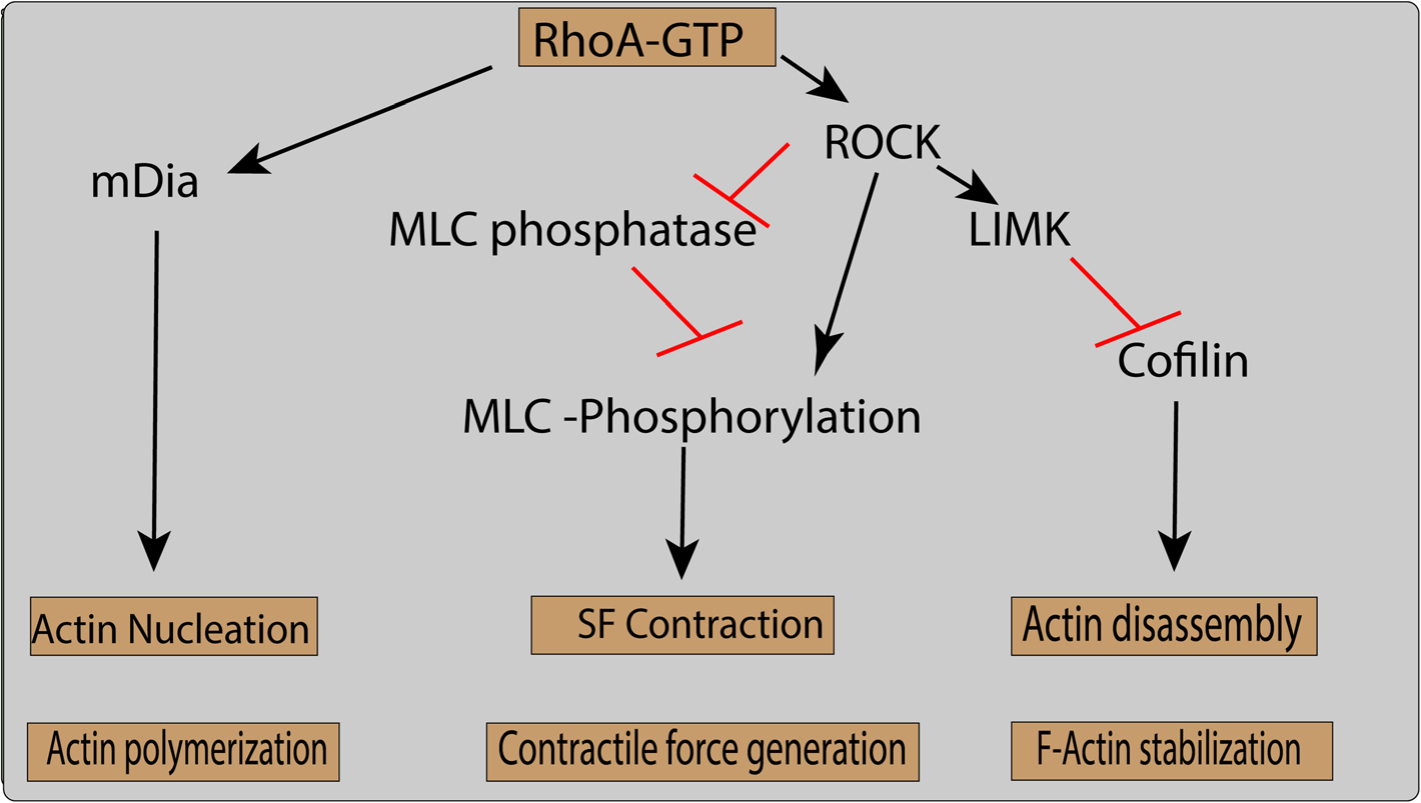
The Rho pathway regulates actin polymerization, contractile force generation, and F-actin stabilization

Rho family GTPase is the key regulatory molecule involved in the remodeling of the actin cytoskeleton. About 20 different types of Rho family proteins, including RhoA, Rac1, and Cdc42, are essential in actin reorganization by interacting with the downstream effector proteins [36, 37]. RhoA is mainly responsible for the generation of cell force and tension within the cell by regulating the activity of myosin II. Activation of RhoA is carried out by mechanical stresses, and inhibition of RhoA or its downstream effectors and mammalian Diaphanous (mDia) and ROCK lead to reorganization of stress fibers [38, 39]. ROCK exist in two forms, i.e., ROCK1 and ROCK2, and both isoforms reported to augment the activity of myosin II. This augmentation is achieved by the phosphorylation of myosin light chain (MLC) either by directly phosphorylating MLC [40] or by indirectly through inhibition of MLC phosphatase (Fig. 2). RhoA and its downstream effectors mediate the association of actin filament and myosin motor molecules which generate actomyosin contractile forces and stress fibers formation [41, 42].

Stress fibers are an actomyosin structure composed of F-actin and myosin-2 held together by crosslinking proteins such as alpha-actinin, fascin, and filamin. Three types of stress fibers are generated in a cell following mechanosensory cues, namely, dorsal stress fibers, transverse arcs, and ventral stress fibers. Dorsal stress fibers only exert a stabilizing function, as they cannot contract [43], and are therefore involved in connecting dorsal stress fibers to transverse arcs [44]. Ventral fibers have abundant concentrations of myosin-2 motors and are connected to focal adhesions. Another fiber subtype, the actomyosin complex, surrounds the nucleus and connects the nuclear envelope to focal adhesions [45]. Direct connections between the nuclear envelope and focal adhesions help the nucleus propagate the mechanical stimuli from focal adhesions into the nucleus [23, 46, 47].

ROCK activates and phosphorylates another kinase, LIM motif-containing protein kinase (LIM kinase), which eventually leads to the phosphorylation of cofilin, an actin-severing protein [41]. Stabilization of actin under tensile force is achieved by inhibiting the activity of cofilin (CFL). LIMK-deficient mice are reported to have reduced bone mass. Additionally, LIMK knockdown results in reduced phosphorylation, which leads to a reduction in osteoblast differentiation and cell viability [48]. A lower level of LIMK and a high level of active (nonphosphorylated) CFL1 were reported during hMSC adipogenic differentiation [49]. LIMK can be phosphorylated by both Rho small GTPase and ROCK, which inactivates CFL1 [50, 51]. Knocking down both CFL1 and Destrin (DSTN) promotes actin polymerization, which in turn facilitates osteoblast differentiation. However, part of DSTN influences osteoblast differentiation mediated partly by enhancing cell viability [23]. The observed difference in both DSTN and CFL1 can be explained by the possibility of different affinities in the binding of actin-binding protein with actin cytoskeleton. Further studies are needed to test this hypothesis.

Several studies have suggested that the abovementioned kinases (Rho, ROCK, Rac, LIMK) might be regulators of osteoblast differentiation [52–54]. These signaling pathways may exert their effects not only through changes in the actin cytoskeleton organization, but also by further signaling through the FAK, JNK, and p38 MAPK pathways [55]. The TGFβ1 pathway is another pathway which regulates the actin cytoskeleton and favors osteogenic MSC differentiation [56].

Numerous factors are involved in regulating actin polymerization and depolymerization. End-to-end polymerization is catalyzed by the formins, whereas secondary branches are promoted by the actin-related protein 2/3 complex (Arp2/3 complex). Blocking the Arp2/3 complex inhibits osteogenesis, which is indicative of the importance of nuclear actin branching in osteogenesis. Inhibition of either mDia2 (found in the cytoplasm) or mDia1 (found in both the cytoplasm and nucleus) results in decreased adipogenesis. In contrast, knocking down mDia2 leads to a decrease in osteogenesis, while silencing of mDia2 has no effect on osteogenesis [57]. Knocking down mDia2 also leads to a decrease in the expression and structure of lamin-B1 [58]. However, it is not clear whether mDia2 knockdown decreases osteogenesis directly through nuclear actin or indirectly through lamin-B1. Surprisingly, a decrease in ossification has been reported after lamin-B1 silencing [59]. In addition to being found at the periphery, lamin A/C and lamin-B1 interact with chromatin in the nucleoplasm [60, 61]. This suggests that mDia2-mediated actin polymerization affects lamin integrity and composition, which might be crucial for MSC differentiation [58].

## Interventions in actin remodeling and their effect on MSC differentiation

### Actin alteration induced by mechanical stresses

The role of mechanical stress-mediated actin alteration and the role of these changes in MSC differentiation have been the subject of numerous studies. Remodeling of actin filaments occurs under static mechanical force, which eventually affects cell proliferation and differentiation. F-actin are aligned under combined static-dynamic stress, whereas irregular actin distribution is observed in the control groups. However, static stress alone did not result in actin filament alignment when compared with dynamic stress. Additionally, hMSC proliferation rates are higher under static stretch as compared to dynamic stretch [62].

The transfer of mechanical signals through the actin cytoskeleton into the nucleus is performed by the LINC complex. Nuclear actin plays an essential role in maintaining the nuclear shape and height during stress. Any disruption to actomyosin contraction results in the alteration of the mechanics inside the cell nucleus. The activity of signaling pathways, such as the Yes-associated protein (YAP) and extracellular-signal-regulated kinase (ERK) pathways, is decreased following a reduction in actomyosin contractility [63]. Tensile stress suppresses adipogenesis [64] and promotes osteogenesis. Cyclic strain increases actin polymerization and longitudinally aligned actin filaments, while a combination of BMP9 with cyclic strain leads to thicker filaments during osteogenesis [65]. Cyclic strain increases cofilin phosphorylation, which helps to stabilize actin filaments through the Rho-ROCK-LIMK pathway [66].

Actin polymerization is regulated by the focal adhesion kinase (FAK) signaling pathway during cyclic stretch which promotes MSC osteogenic differentiation [67]. Actin polymerization and the bundling of stress fibers are reinforced by the activation of mammalian target of rapamycin complex 2 (mTORC2); as a result, actin polymerization favors osteogenesis [68]. Compression forces reduce the height of cells and circumferentially aligned stress fibers. An increase in the contractility was observed by these stress fibers, which eventually results in resistance to the mechanical force [69]. Compression forces on hydrostatic collagen gels induce a drastic change in cell morphology, whereby cells become flattened and completely lack stress fibers. However, cells in compressed gel develop an ovoid shape and present a close network of numerous actin filaments. The actin filaments in dense gels show a greater tendency toward osteogenic differentiation as compared with hydrostatic gels [70]. Another study showed a similar result for F-actin stress fibers, which become prominent when estrogen was added under mechanical pressure, wherein, alkaline phosphatase activity, an early indicator of osteogenesis, was observed to be higher showing osteogenesis [71].

Fluid flow shear stress promotes robust actin polymerization and facilitates osteogenesis. Fluid shear stress induces Rho activation, which is imperative for the nuclear translocation of the transcriptional co-activator with PDZ-binding motif (TAZ) transcription factor [72]. Fluid shear stress increases osteogenesis by increasing calcium influx and F-actin assembly. A correlation exists between fluid shear stress-induced osteogenesis and F-actin [73]. F-actin can mediate the assembly and disassembly of intermediate filaments and vinculin (involved in focal adhesion), which are required for the induction of osteogenesis through the transient receptor potential melastatin 7 (TRPM7)-osterix axis. This suggests that F-actin might indirectly act as a mediator in fluid shear stress-induced osteogenic differentiation [73]. Further studies are required to completely understand this process.

Microgravity has been used to study physiological changes, such as the maintenance of MSCs in the undifferentiated state and cell proliferation and differentiation [74, 75]. The most important change that occurs inside the cell is the disruption of actin filaments through impaired Rho signaling. Sustained stimulation by microgravity promotes adipogenesis during MSC differentiation [76, 77]. Osteogenesis is inhibited as actin depolymerization prevents the translocation of transcriptional co-activator with PDZ-binding motif (TAZ) into the nucleus [55]. Sinusoidal vibration has a role in the formation of F-actin fibers, which ultimately results in osteogenic MSC differentiation. The exact mechanism underlying the vibration-induced differentiation is not yet clear, and further studies are required to explore this molecular phenomenon [78]. Low-intensity vibration leads to the disruption of actin fibers, which favors adipogenesis in MSC differentiation; however, the exact mechanism is not yet clear [79]. An ultrasound-based method, acoustic tweezing cytometry, also facilitates the formation and contractility of F-actin, which ultimately promotes Yes-associated protein (YAP) translocation, thereby favoring osteogenesis [80].

### Chemical clues induce actin modifications

Following chemical intervention, cells undergo a drastic transition from polymerization to depolymerization and vice versa [81]. F-actin and G-actin both play a vital role in the osteogenic and adipogenic differentiation of MSCs. During adipogenic differentiation, the ratio of G-actin to F-actin is increased (76% on day 13), whereas the F-actin fraction is higher during osteogenic differentiation [49, 55]. Cell stiffness varies in a manner that is dependent on the polymerization and depolymerization of actin filaments. Studies have demonstrated that depolymerization decreases cell rigidity that eventually favors chondrogenesis or adipogenesis, whereas actin polymerization promotes MSC commitment to an osteogenic fate [16, 17, 29, 82].

Chemical induction of actin polymerization and depolymerization influences the differentiation of MSCs. Disruption of the cytoskeleton by treatment with cytochalasin D (CD) leads to a significant decrease in the levels of osteogenic markers, i.e., calcium deposition and alkaline phosphatase (ALP), when compared with untreated cells. Additionally, a change in the cytoskeleton at the initial stages of differentiation is sufficient to affect the levels of osteogenic markers. This suggests that actin cytoskeleton integrity is essential for MSCs to show the phenotypic behavior of differentiated cells [29].

**Fig. 3:**
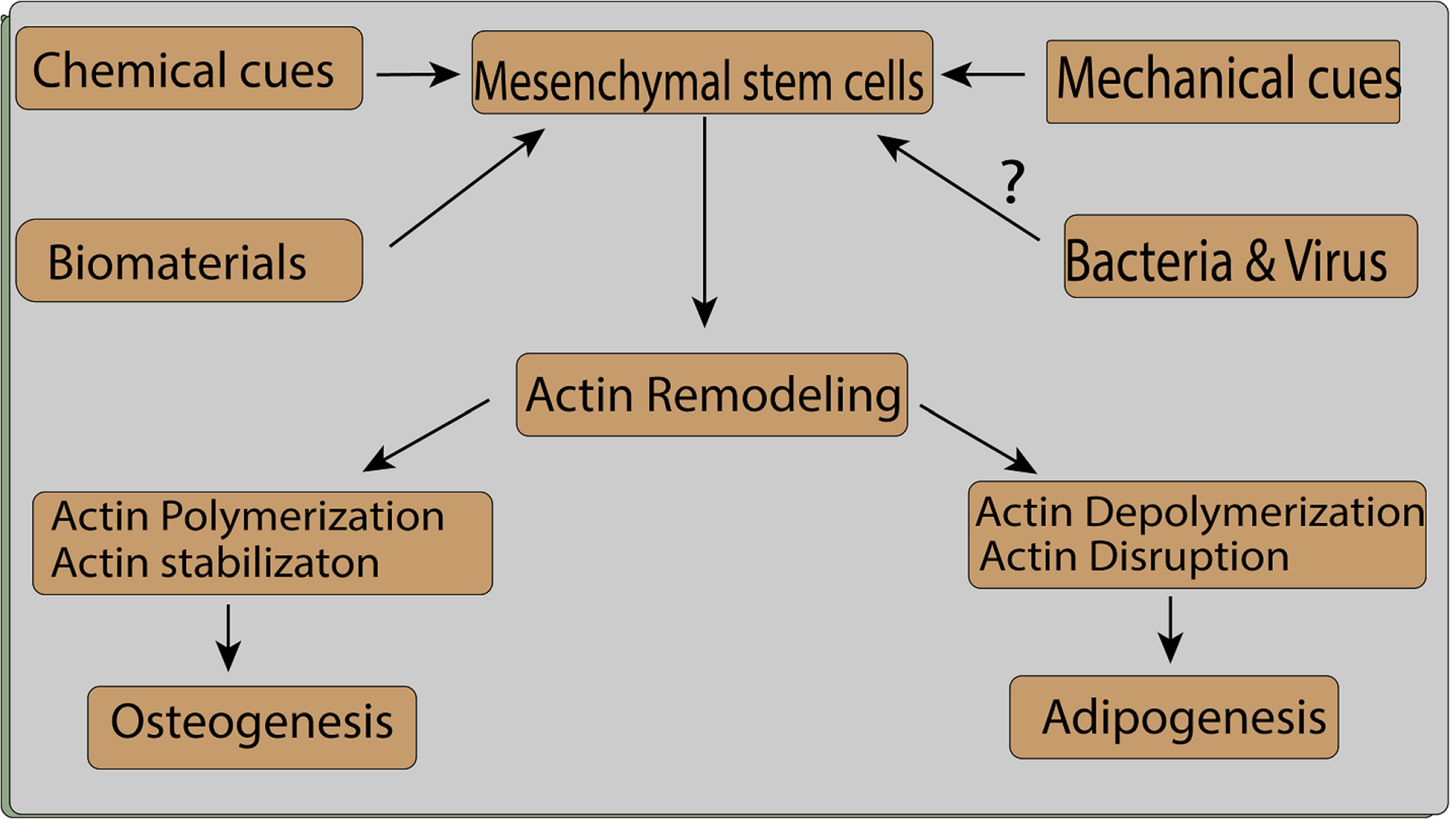
Mechanical, chemical, biomaterial, and possible pathogen-related interventions lead to actin reorganization and facilitate osteogenesis or adipogenesis

Numerous studies have suggested that an inverse correlation exists between actin polymerization and adipogenesis, whereas there is a direct correlation between actin polymerization and osteogenesis (Table 1) [49]. CD treatment has been reported to reduce actin depolymerization, which leads to osteogenic and adipogenic differentiation in hMSCs. In vivo injection of CD contributes to an increase in bone mass and adipocyte generation [84] (Fig. 3). This result was different from those of other studies and can be attributed to cellular composition or the duration of CD treatment. Actin polymerization may be downregulated at the initial phase of differentiation [49, 84]. Sen et al. [72] reported the increased availability and translocation of G-actin into the nucleus, resulting in increased expression of osteogenic and adipogenic-related gens [84]. Several studies have indicated that cytoplasmic F-actin branching is increased during osteogenic differentiation [16, 17, 35, 49, 55]. A different study, using RNA-Seq analysis, reported that CD treatment induces osteogenesis via the vestigial-like family member 4 (*VGLL4*) gene and that the effect of CD depends on the biological state of the cells analyzed [85]. This effect of CD on different tissue needs to be further elucidated to explain the role of CD effect on different stem cell types and their differentiation.

**Table 1:** Chemicals that promote actin polymerization or depolymerization and their role in the osteogenic or adipogenic differentiation of mesenchymal stem cells

Chemical	Dose and duration	Osteogenic/adipogenic marker	References
Cytochalasin D	0.1 μg/mL for 48 h	ALP and calcium levels decreased at days 5 and 10	[29]
Cytochalasin D	100 ng/mL for 1, 3, 7, and 14 days	Decreased levels of ALP and osteocalcin, increased levels of adiponectin and peroxisome proliferator-activated receptor gamma (PPARG)	[16]
Cytochalasin D	0.02 mg/mL	Increased adipogenesis	[83]
Cytochalasin D	1–20 μM, 1 h every day for 9 days	Decreased osteoblast differentiation, decreased ALP and mineral matrix	[55]
Phalloidin	0–6 μM, 3 h every day for 9 days	Increased ALP activity and mineralized matrix formation	
Cytochalasin D	0.1 mg/mL day 1	Osterix, osteocalcin, and Runt-related transcription factor 2 (RUNX2) levels increased on days 2 and 3	[84]
Cytochalasin D	0.1 mg/mL for 3 days	Increase in osteogenesis (higher level of alkaline phosphatase, tissue-nonspecific isozyme (Alpl), specificity protein (Sp7), gamma-carboxyglutamic acid-containing protein (Bglap genes)) and adipogenesis (higher level of fatty acid-binding protein (FABP4), adiponectin gene (ADIPOQ), and peroxisome proliferator-activated receptor γ (PPARγ genes)) in growth medium, increased adipogenesis in adipogenic medium, and increases osteogenesis in osteogenic medium	[57]
Cytochalasin D	0.1 mg/mL for 14 days	Increase in osteogenesis through increased expression of the *VGLL4* gene; the effect of cytochalasin D was dependent on the biological state of the cells	[85]
Cytochalasin D	1–20 μM for 1 h every day for 13 days	Increased adipocyte differentiation	[49]
Phalloidin	0–3 μM for 3 h every day for 13 days	Decreased adipocyte differentiation and adipocyte-specific gene expression (*ADIPOQ*, *LPL*, *PPARG*, *FABP4*)	[49]
Cytochalasin D	–	Increased adipogenesis through the regulation of the *FGF2*, *TGFβ2*, *EGR2*, *MEF2D*, and *IRS1* genes	[56]

Shuttling of G-actin between the cytoplasm and the nucleus is a highly regulated process [86]. A threefold increase in G-actin was observed in the nucleus after treatment with CD, which led to reduced levels of cytoplasmic actin. Actin is translocated into the nucleus with the help of importin 9 and cofilin [57, 87] and is reported to be the trigger for osteogenesis in MSCs. Knocking down cofilin and importin reduces actin shuttling into the nucleus, which eventually suppresses the osteogenic process. Actin has also been reported to have a role in gene expression, through influencing chromatin remodeling, RNA processing, and transcription [88]. Nuclear actin has been suggested to be directly involved in MSC differentiation into different lineages.

**Fig. 4:**
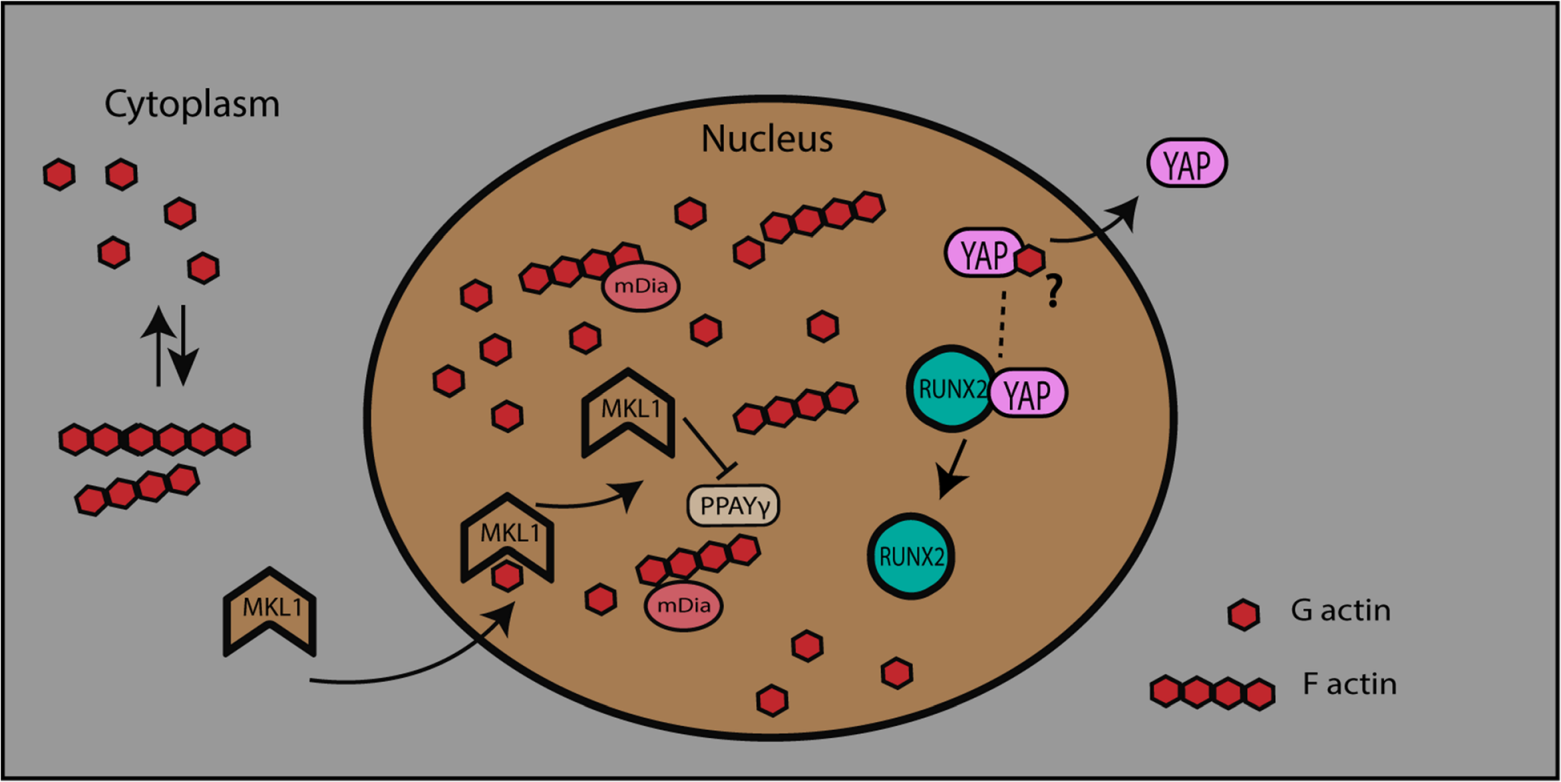
Actin facilitates the movement of MKL1 into the nucleus and nuclear YAP exclusion, which regulates differentiation

Nuclear actin-induced osteogenic differentiation might depend on the availability of the YAP transcription factor. Actin depolymerization in the cytoplasm results in the nuclear influx of G-actin that subsequently leads to YAP exclusion from the nucleus. Studies have shown that RUNX2 expression is repressed through its binding to YAP [89], wherein YAP was translocated out of the nucleus by the influx of G-actin [57]. Nuclear YAP exclusion is associated with reduced proliferation [90] which may subsequently also affect differentiation [91]. Similarly, an increase in the G-actin/F-actin ratio is observed in adipogenic differentiation media. G-actin also binds to megakaryoblastic leukemia 1 (MKL1) in the cytoplasm and prevents its translocation into the nucleus, which results in an increase in adipocyte differentiation. An antagonistic relationship exists between PPARG and MKL1 in adipocyte differentiation, whereby knockout of MKL1 leads to an increase in white adipogenesis (Fig. 4) [92]. A different study indicated that MKL1 and serum response factor (SRF) independently negatively regulate brown adipogenesis [93]. Nuclear G-actin polymerization may be required for the initiation of MSC differentiation, an idea that requires further investigation. The inner nuclear membrane-localized protein lamin A/C and emerin might have a regulatory role in actin polymerization [94] during the initiation of differentiation. Actin depolymerization is a key regulator of adipogenesis during MSC differentiation. Actin depolymerization increases the levels of phosphorylated p38 and ERK1/2 and also increases the gene expression of *PPARG* during adipogenesis [83]. Similar findings have been reported in another study, which showed that adipogenic and osteogenic differentiation is regulated by the p38 MAPK and ERK1/2 pathways through the remodeling of actin filaments [16].

## Interventions in actin remodeling and their effect on MSC differentiation

### Biomaterial induced actin remodeling

In addition to the external mechanical forces on cells mentioned above, intracellular forces are shifted to cells through ECM adhesion or by cell-cell junctions. The stiffness of intrinsic forces is proportional to the stiffness of the matrix [95]. Intracellular pathways are also influenced by these forces, which eventually change the expression of genes and proteins through nuclear signaling proteins. Integrins are the cell components which make up the connection between cell and outer environment, and these integrins trigger the cell-ECM interaction [96–98]. The association of extracellular components with the cytoskeleton is carried out through the cytoplasmic domain of integrins forming the focal adhesion zone. These adhesion sites are composed of adhesome (protein complexes) which allow mechanical coupling [99–104]. Moreover, assembly and disassembly of adhesome are affected by substrate elasticity. In fact, soft substrate destabilizes the adhesion, whereas stiff substrate produces forces which in turn adhesion stabilization [105–107].

The cell activates myosin II motors when it interacts with the substrate, which leads to the changes in the confirmation of adhesome’s components such as talin, vinculin, and focal adhesion kinase FAK [95]. The force from ECM to cell modulates focal adhesion and FAK signaling pathway, thereby activating the RhoA signaling by phosphorylating it [108]. RhoA then augments the activation of ROCK, which eventually phosphorylates MLC. Thus, through local adhesome, intracellular actin cytoskeleton senses the cell force results in the activation of multiple mechanosensitive pathways, including YAP/TAZ and MKL1 [109, 110].

Stem cells assess substrate stiffness which encourages its differentiation into different lineages. Neural differentiation is favored in soft substrate, whereas moderate substrate stiffness appreciates chondrogenic differentiation. Similarly, the stiffer substrate encourages osteogenic differentiation in stem cells [111–113]. These results suggested that the elastic modulus of native tissue is similar to the elastic modulus of stem cell differentiation; thereby, osteogenic differentiation is favored when a stiff scaffold is used [114]. Besides, various new microenvironments such as 2D and 3D scaffolds are also utilized to assess the differentiation potential of stem cells. Morphological structure, gene expression, and cell function, proliferation, and differentiation of cells in 3D are different than the cells in 2D [115]. Furthermore, the 3D scaffold can be able to augment the production of osteogenesis [116] and chondrogenesis as compared to 2D scaffold and thus is being used more frequently in osteochondral tissue engineering [117, 118].

Stem cell behavior is influenced by the surface topography as well and thereby, survival of stem cells is based on the topography of scaffold, in vivo [119]. However, in vitro, surface topography influences cell adhesion, gene expression, proliferation, and fate of stem cells. Roughness and texture are the two most important properties of surface topography which help in the regulation of cell fate. On a rough surface, the proliferation of stem cells was reported to be reduced and osteogenesis was favored. Moreover, concave surfaces showed better interaction of stem cells with more inclination toward osteogenesis [120]. Recently, nanomaterials are emerged as potential candidates in the development of biomaterial to influence stem cell fate. In fact, their small size and bioactive characteristics made them more suitable to interact with physiologic environment of cells. Nanomaterial (NM) composition, topography, and morphological and electrical properties have been shown to influence stem cell response [121]. Bacakowa et al. studied 3 different layers of nanoparticle coated with fullerene C60, single-wall carbon nanotubes (SWCNTs), and multiwall carbon nanotubes (MWSNTs). The actin filament turned into a thick bundle and cells spread more than control [122]. Another research recently proposed the interaction of nontopographic structures with actin cytoskeleton which result in actomyosin contraction. This contraction affects cell migration and cell spreading in the 3D environment [123]. Various types of nanomaterial and their effect on the differentiation of MSCs will be discussed in Table 2.

**Table 2:** Nanomaterial’s use in mesenchymal stem cells

Nanomaterial		Differentiation potential	
Polymeric NMs	Poly(d,l-lactide-*co*-glycolide)-bovine albumin serum (PLGA-BSA)	Increase osteogenesis, decrease adipogenesis differentiation	[124]
Ceramic NMs	Hydroxyapatite nanoparticles (HAP NPs (20 nm))	Enhance osteogenic differentiation	[125]
Carbon NMs	SWCNTs	Enhance adipogenic, osteogenic	[126]
Carbon NMs	Reduced graphene oxide (rGO) nanosheets	Enhance osteogenic differentiation	[127]
Metal/metal oxide NMs	Chitosan-gold nanoparticle (AuNPs)	Enhance osteogenic differentiation	[128]
Metal/metal oxide NMs	BSA-AuNPs (70, 100 nm)	Enhance osteogenic differentiation	[129]
Metal/metal oxide NMs	BSA-AuNPs (40 nm)	Decrease osteogenic differentiation	[129]
Metal/metal oxide NMs	BSA-coated gold nanorods (70 nm)	Enhance osteogenic differentiation	[129]
Metal/metal oxide NMs	Polyethylene glycol-gold nanoparticles PEG-AuNPs (4 nm)	Decrease osteogenic differentiation	[130]
Metal/metal oxide NMs	Gold nanoparticles, carboxylic acid (AuNP–COOH) (17 nm)	Decrease osteogenic differentiation	[131]
Metal/metal oxide NMs	Zincoxide (ZnO NPs)	Enhance osteogenic differentiation	[132]

### Pathogen induced actin alteration

The pathogen can take a wide variety of beneficial outcomes after manipulating the host cells, for example, actin filament formation at the apical surface of mucosal epithelia by activating Rac and Cdc42. This activation is achieved after translocating effectors *Salmonella typhimurium* exchange factor (SopE and SopE2) into host cells [133–136]. F-actin formation results in the membrane ruffling which causes an intake of the pathogen inside of cells by micropinocytosis [133, 137, 138]. Other gram-positive bacteria *Listeria* spp. cause actin polymerization on its own surface using the activity of protein ActA which is an analog of members of the Wiskott-Aldrich syndrome protein (WASP) nucleation promotion factor. Branched actin polymerization is achieved on the surface of a pathogen by recruiting the Arp2/3 complex which helps bacteria to move into the cells. There are various bacteria mentioned in Table 3 which induce polymerization [139, 140]. Some bacteria, i.e., *Escherichia coli*, attach to the cell membrane by structuring specialized actin filaments [141], whereas others, i.e., *Chlamydia*, induce actin polymerization in host cells helping itself for its reproduction [143, 144].

**Table 3:** Pathogens and actin interaction

Pathogen	Mechanism of action adaptation in host cell	References
*Salmonella* spp.	Translocate effectors (SopE and SopE2) into host cells which increase F actin polymerization.	[133, 134, 137]
*Listeria monocytogenes*	ActA protein recruits an Arp2/3 on the surface of listeria which promotes actin polymerization that helps in the movement of bacteria in the cells.	[139, 140]
*E. coli*	Actin-rich filament that facilitates their attachment.	[141, 142]
*Chlamydia trachomatis*	Secrete actin-recruiting phosphoprotein (Tarp) which cause actin polymerization depolymerization in the host cell.	[143, 144]
*Coxiella burnetii*	Infects phagocytic human macrophages via binding to complement receptor 3 (CR3) receptors, triggering the reorganization of filamentous actin at the attachment site.	[145]
*Rickettsia conorii*	Attachment to host cell requires actin rearrangement via recruitment and activation of Arp2/3.	[146]
Tick-borne pathogen *Anaplasma phagocytophilu*	Actin polymerization at invasion.	[147]
*Ehrlichia chaffeensis*	Manipulation of cytoskeleton through SUMOylation-dependent protein-protein interactions between bacterial effectors and host cytoskeletal components.	[148]
*Vaccinia* viruses	Receptor tyrosine kinase signaling which in turn ignite actin polymerization through N-WASP-Arp2/3 cascade.	[149]

The role of these bacteria and viruses on actin polymerization was not studied on MSC differentiation. However, recently, a study on heat-inactivated bacterial pathogens *Escherichia coli*, *Staphylococcus aureus*, and *Streptococcus pyogenes*, showed to have increased MSC proliferation and differentiation [150]. Moreover, a balanced approach of probiotics and oral bacteria is proved to increase the MSC proliferation and osteogenic differentiation [151]. Modification in the actin cytoskeleton during the interaction of certain bacteria and viruses with the cell could be a potential area for the induction of MSC differentiation. Application for advanced techniques like gene editing and bioengineering, etc. in a bacterial cell by making it less pathogenic [152] eventually could help reprogram the MSCs cells into multiple lineages. Moreover, it could help in regeneration medicine and cell-based therapy using actin remodeling as the main tool against infectious diseases.

## Conclusion

Actin filaments appear to be a vital determinant in the differentiation of MSCs. Actin remodeling is a commonly observed phenomenon during the differentiation of MSCs into adipogenic and osteogenic lineages. Actin polymerization, stabilization, and stress fiber formation are observed in osteogenesis, whereas less organized and depolymerized actin networks are observed during adipogenesis. The literature reviewed here suggested that actin polymerization and depolymerization appear to be an essential element for osteogenesis and adipogenesis, respectively. Not surprisingly, osteogenic cells appear stiffer which have stabilized actin fibers, whereas disrupted actin was found in round adipogenic cells. Various interventions, i.e., mechanical, chemical, and biomaterial, also suggested actin polymerization and stabilized actin in osteogenic cells as compared to actin depolymerization in adipogenic cells. In addition, pathogens induce actin polymerization for their invasion and use actin machinery of host cells which might be taken as a tool for regenerative medicine and cell-based therapy. However, there are few questions unanswered; how actin perceives varied signals from the environment and decides to create softness and stiffness in the form of cell tension within the cells?

Although chemically induced actin depolymerization favors adipogenesis, future studies should focus on the role of actin polymerization/depolymerization in cell differentiation. However, it is essential to understand how actin contributes to osteogenesis and what is the role of nuclear actin in cell differentiation. In addition, further studies are required to determine the role of signaling pathways that regulate actin organization, such as TGFβ1, in MSC differentiation. TGFβ1 mediates actin cytoskeleton organization during osteogenesis; however, TGFβ1 or Cyto D treatment alone also induce adipogenesis. Therefore, TGFβ1 or Cyto D might either promote different patterns in actin filament, or actin cytoskeletal reorganization might be independent of TGFβ1 [56]. Furthermore, a better understanding of how the balance between YAP and TAZ is regulated and how this balance is affected by the cytoskeleton is also required.

## Data Availability

Data sharing is not applicable to this article as no datasets were generated.

## Abbreviations

MSCs: Mesenchymal stem cells
ECM: Extracellular matrix
Arp2/3 complex: Actin-related protein 2/3 complex
Rho: Ras homologous
ROCK: Rho-associated coiled-coil-containing protein kinase
MLC: Myosin light chain phosphorylation
Cyto D: Cytochalasin D
mDia: Mammalian diaphanous
YAP: Yes-associated protein
ERK: Extracellular signal-regulated kinase
LIMK: LIM motif-containing protein kinase 1
mTORC2: Mammalian target of rapamycin complex 2
TAZ: Transcriptional co-activator with PDZ-binding motif
TRPM7: Transient receptor potential melastatin 7
ALP: Alkaline phosphatase
*VGLL4*: Vestigial-like family member 4
RUNX2: Runt-related transcription factor 2
SP7: Specificity protein
BGLAP: Bone gamma-carboxyglutamate protein
FABP4: Fatty acid-binding protein 4
ADIPOQ: Adiponectin gene
PPARγ: Peroxisome proliferator-activated receptor γ
FGF2: Fibroblast growth factor 2
TGFβ2: Transforming growth factor-β2
EGR2: Early growth response 2
*MEF2D*: Myocyte enhancer factor
IRS1: Insulin receptor substrate 1
CFL1: Cofilin-1
CFL2: Cofilin-2
MKL1: Megakaryoblastic leukemia 1
sopE: *Salmonella typhimurium* exchange factor
Tarp: Secrete actin-recruiting phosphoprotein
CR3: Complement receptor 3
ActA: Activity of protein
WASP: Wiskott-Aldrich syndrome protein
ZnO: Zincoxide
PEG-AuNPs: Polyethylene glycol-gold nanoparticles
AuNPs: Gold nanoparticle
rGO: Reduced graphene oxide
HAP NPs: Hydroxyapatite nanoparticles
PLGA-BSA: Poly(d,l-lactide-co-glycolide)-bovine albumin serum
SWCNTs: Single-wall carbon nanotubes
SRF: Serum response factor
